# Neuroscience in Youth Criminal Law: Reconsidering the Measure of Punishment in Latin America

**DOI:** 10.3389/fpsyg.2020.00302

**Published:** 2020-02-25

**Authors:** Nicolás Ezequiel Llamas, José Ángel Marinaro

**Affiliations:** Department of Law and Political Science, National University of La Matanza, San Justo, Argentina

**Keywords:** neurolaw, youth criminal law, juvenile crime and delinquency, juvenile criminal behavior, juvenile criminal law, capital punishment, life imprisonment, life imprisonment without parole

## Introduction

Due to the new discoveries and advances made in technology in the field of neuroscience in the last few decades, it has been possible to get a better understanding of the development of the human brain. This has had a significant impact on youth criminal law, especially in relation to the behavior of adolescents and their capacity to control impulsive reactions.

In this article, we will discuss the repercussions of this improved understanding on the amount of penalty for convicted adolescents in Latin America.

It is important to mention that the minimum age of criminal responsibility on each country of this region is quite different (mostly between 12 and 16 years old). Despite this and other divergences, we think it is possible to make an approach from the point of view of the Inter-American Human Rights System.

## Measure of Punishment: Comparative Disproportion

It could be argued that the majority of actions or omissions which constitute a crime in a certain country usually also constitute a crime in most countries around the world. However, the measure of the punishment that could be imposed as result of that same crime does not follow this generalization. In this respect, for example, there are several countries that do not impose capital punishment or life imprisonment.

**Figure 1:**
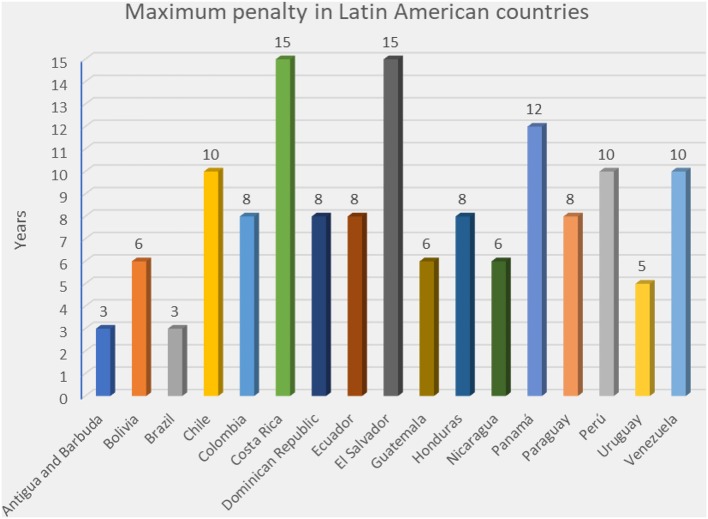
This figure shows the maximum penalty in some Latin American countries that may be imposed on adolescents (as per references listed below). Countries that allow life imprisonment or capital punishment have been excluded for not been able to be shown. Antigua and Barbuda: *Child Justice Act* (No. 23 of 2005), c. X, s. 69(2); Bolivia: Law 548 [*Código Niña, Niño y Adolescente*], s. 268; Brazil: Law 8069 [*Estatuto da Criança e do Adolescente*], s. 121; Chile: Law 20084 [*Sistema de Responsabilidad de los Adolescentes por Infracciones a la Ley Penal*], s. 18; Colombia: Law 1098/2006 [*Código de la Infancia y la Adolescencia*], s. 187; Costa Rica: Law 7576 [*Ley de Justicia Penal Juvenil*], s. 131; Dominican Republic: Law 136-03 [*Código para la protección de los derechos de los Niños, Niñas y Adolescentes*], s. 340; Ecuador: Law 100 [*Código de la Niñez y Adolescencia*], s. 358(3); El Salvador: Law 869 [*Ley del Menor Infractor*], s. 15-17; Guatemala: Law 27/2003 [*Ley de Protección integral de la niñez y adolescencia*], s. 252(b); Honduras: Law 73/96 [*Código de la Niñez y la Adolescencia*], s. 205; Nicaragua: Law 287/1998 [*Código de la Niñez y la Adolescencia*], s. 206; Panamá: Law 40/1999 [*Régimen Especial de Responsabilidad Penal para la Adolescencia*], s. 141; Paraguay: Law 1680/2001 [*Código de la Niñez y la Adolescencia*], s. 207; Perú: Law 27337 [*Código de los Niños y Adolescentes*], s. 235; Uruguay: Law 17823 [*Código de la Niñez y la Adolescencia*], s. 91; Venezuela: Law 5859 [*Ley Orgánica para la Protección del Niño, Niña y Adolescente], s. 628*. Canada (*Youth Criminal Justice Act* [S.C. 2002, c. 1, s. 64(1)] and Grenada [*Juvenile Justice Act*, Act No. 24 of 2012, s. 4(2)] may impose life imprisonment. Through the reports called “Concluding observations” made by the United Nations Committee on the Rights of the Child (available on https://www.ohchr.org/SP/Countries/LacRegion/Pages/LacRegionIndex.aspx) we were able to establish that the death penalty could be imposed in Bahamas and Saint Lucia; and life imprisonment could be imposed in Barbados, Belize, Cuba, Dominica, Guyana, Haiti, Jamaica, Mexico, Saint Kitts and Nevis, Saint Vincent and the Grenadines, Suriname and Trinidad and Tobago.

In this context, and according to Comparative Law, we find large disparities between the penalties applied in different countries by the youth criminal law, the body of law that regulates crimes committed by a person under the age of majority. Latin American countries are a great example of this situation: while Brazil has a maximum penalty of 3 years of imprisonment for any crime committed by an adolescent between 12 and 18-year-old (Law 8069 [*Estatuto da Criança e do Adolescente*], s. 121), other countries, like Bahamas [*Penal Code*, s. 263 (3)] allow capital punishment. More examples are shown in Figure 1.

In order to analyze this correctly, we propose to classify the different legislative methods into three groups. First, there are legal systems that allow the transfer of young offenders to a criminal court (also known as “trial as an adult”). Second, there are those that allow the juvenile court to impose an adult sentence. Third, there are those that only allow juvenile sentences for young offenders, which are considerably less severe than adult sentences.

The first method is common in countries that have adopted the legal system known as Common Law (pure or mixed). The decision to transfer a young offender may contemplate several factors, but the most important ones are the severity of the offense and the age of the offender. This decision may be made by a judge (judicial waiver), a prosecutor (prosecutorial discretion), or by the law itself (statutory exclusion).

The second method is mostly used in countries that have adopted the civil law system. Like the prior one, the severity of the offense and the age of the offender are the main factors used to make the decision. Despite their differences, both systems enable the sentencing of a young offender as an adult.

The third one, however, prohibits that kind of penalty, which also means it prohibits capital punishment and life imprisonment. It is not possible to make any other assumption about this matter since the maximum amount of penalty is quite diverse in every jurisdiction.

Additionally, there are international laws that prohibit capital punishment or life imprisonment for young offenders, like article 37 of the United Nations Convention on the Rights of the Child, and human-rights courts that do not allow for adolescents to be sentenced with the same punishment that may be imposed on an adult, like the leading case “Mendoza et al. v. Argentina, Preliminary Objections, Merits, and Reparations, Judgment” of the Inter-American Court of Human Rights (ser. C, No. 260, May 14, 2013).

## Overview of Neurolaw Regarding Adolescents

Having said all that, it should be affirmed that several neuroscientific studies have proved that adolescents do not have the same cognitive capacity as an adult. In particular, it has been suggested that the frontal lobe, whose functions involve controlling and judging impulse and risk, projecting future consequences resulting from current actions (Fuster, 2001; Martinez Selva et al., 2006), continues its development well into young adulthood (Gogtay et al., 2004; Giedd, 2008).

Thus, disadvantageous decision making and risky behavior shown by adolescents are considered to be related to the slower developing prefrontal cortex (Smith et al., 2012), which has been linked to prominent differences in cognitive capacity (Cauffman and Steinberg, 2000; Galvan et al., 2006; Eshel et al., 2007). Further investigations have been made, some of them related to drug abuse or peer influence, which support this matter (Blakemore, 2012; Spear, 2013; Brizio et al., 2015; van Duijvenvoorde et al., 2016)^1^.

The impact of those studies was meaningful for the judiciary system of the United States since they were used by its Supreme Court to sentence the leading cases *Roper v. Simmons* (543 U.S. 551), *Graham v. Florida* (560 U.S. 48), and *Miller v. Alabama* (567 U.S. 460). In addition, there is an ongoing debate about their legal implications (Steinberg, 2009; Delmage, 2013).

## Discussion

The Supreme Court of the United States stated that “a sentence lacking any legitimate penological justification is by its nature disproportionate to the offense” (*Graham v. Florida*, 560 U.S. 48, p. 20). However, it is necessary to analyze if penological justifications designed for adults are applicable to juveniles.

This implies a change in basic assumptions. Penological justifications have been created and built on suppositions tied up with notions of agency, freedom, and free will. Whenever a sentence requires a person acting purposely, the lack of intent means there is absence of blameworthiness as well as absence of any justification for condemning. Therefore, if it is proved that adolescents do not have the same capacity as an adult to observe the law, it does not only impact on the personal culpability but also the assumptions of the penological justification itself.

In this regard, equality and non-discrimination before the law should not only be considered as giving the same legal treatment to all human beings in general, but also to give different treatment to those who are not equals. Consequently, applying similar punishment to juvenile offenders and adult offenders for the same crime should be judged incompatible with legal principles and also with the current state of the science.

In modern criminal law there is no debate that any sentence must take into consideration the moral responsibility of the perpetrator. However, this same principle wrongly causes controversy when the outcome of its application consists of a reduction in culpability, and therefore in the size of the imposed penalty.

For all these reasons, we consider that any law or jurisprudence that makes the transfer of a juvenile offender to an adult court possible, or allows an adult sentence to be imposed on them, ought to be reconsidered. As it was mentioned before, there are many countries whose legislation provides considerable differences between juvenile and adults offenders, and the Inter-American Court of Human Rights has represented an important step in this direction (Llamas, 2019).

Nevertheless, the legal impact of the neuroscientific findings and technologies is an open debate (Muñoz Ortega, 2013, 2018). Nowadays, we are observing an exponential increase of publications about adolescents and their behavior related to alcohol, drugs, stress, and peer influence, among other topics. Some of them even suggest that the age of 18 is not a scientifically correct watershed between adolescent and adult criminal responsibility (Mercurio, 2012; Mercurio et al., 2019).

The topic is crucial when considering some countries with high levels of poverty and malnutrition in childhood, which may affect the development of the human brain and its cognitive abilities (Mercurio, 2016), as well as the known effects of deprivation (Llamas and Marinaro, 2017)^2^.

As a final reflection, we want to mention that some very old Spanish laws, which were in force long before the independence of Latin-American countries (López de Guevara, 1843), did not allow adolescents to be sentenced as adults. In a way, it seems that new discoveries might prove scientifically what was presumed righteous long ago.

## Author Contributions

NL and JM wrote the manuscript with equal contributions.

### Conflict of Interest

The authors declare that the research was conducted in the absence of any commercial or financial relationships that could be construed as a potential conflict of interest.

## Footnotes

^1^There are other aspects of the development of prefrontal cortex which might play a major role in terms of behavioral outcomes, such as hormonal influences onto the brain (Blakemore et al., 2010).

^2^It is important to remark that there are cultural and ethical non-scientific aspects that were at the basis of the exclusion of the most severe punishments for young people in Europe and in the United States.
